# The effect of cardamom supplementation on serum lipids, glycemic indices and blood pressure in overweight and obese pre-diabetic women: a randomized controlled trial

**DOI:** 10.1186/s40200-017-0320-8

**Published:** 2017-09-29

**Authors:** Yaghooblou Fatemeh, Fereydoun Siassi, Abbas Rahimi, Fariba Koohdani, Farideh Doostan, Mostafa Qorbani, Gity Sotoudeh

**Affiliations:** 1 Department of Community Nutrition, School of Nutritional Sciences and Dietetics, Tehran University of Medical Sciences, Hojatdost street, Naderi street, Keshavarz Blv, Tehran, Iran; 2 Department of Epidemiology and Biostatistics, School of Public Health, Tehran University of Medical Sciences, poursina street, Keshavarz Blv, Tehran, Iran; 3 Department of Cellular and Molecular Nutrition, School of Nutritional Sciences and Dietetics, Tehran University of Medical Sciences, Tehran, Iran; 4 Department of Nutrition, School of Health, Kerman University of Medical Sciences, Kerman, Iran; 5 Non-communicable Diseases Research Center, Alborz University of Medical Sciences, Karaj, Iran

**Keywords:** Cardamom supplementation, Pre-diabetes, Glycemic indices, Anthropometry, Women

## Abstract

**Background:**

Spice consumption helps the treatment of diseases due to their antioxidant and anti-inflammatory contents. Cardamom is one of this spices; therefore, this study is designed to determine the effect of cardamom supplementation on serum lipids, glycemic indices, and blood pressure in pre-diabetic women.

**Methods:**

Eighty overweight or obese pre-diabetic women were randomly allocated to two groups. The intervention group received 3 g of green cardamom and the placebo group received 3 g of rusk powder for 2 months. The physical activity level, dietary intake, anthropometric measurements, Blood pressure, fasting blood sugar (FBS), triglyceride (TG), total cholesterol (TC), low density lipoprotein (LDL-C), high density lipoprotein (HDL-C), insulin, body mass index (BMI), insulin resistance, and insulin sensitivity were measured before and after intervention.

**Results:**

After intervention, mean TC (*p* = 0.02) and LDL-C (*p* = 0.01) significantly decreased and insulin sensitivity (*p* = 0.03) increased in the cardamom group. In the control group, mean HDL-C (*p* = 0.02) significantly decreased after the study. We observed no significant decrease in systolic and diastolic blood pressure, glycemic indices, and serum lipids values in the cardamom group compared to the placebo group.

**Conclusions:**

Green cardamom supplementation may have a protective effect on HDL-C level in pre-diabetic subjects. It improves some blood parameters in these subjects; however, its effects are not different from placebo.

**Trial registration:**

Iranian Registry of Clinical Trials, IRCT2014060817254N2. Registered 2 September 2014.

## Background

Diabetes is one of the world’s health challenges and its detection in an early stage can reduce economic burden of the disease on society [1]. Population growth, urbanization, increasing prevalence of obesity, and reduced physical activity have caused a rising prevalence of diabetes and pre-diabetes worldwide [2, 3]. Identifying pre-diabetes and changing the lifestyle help in delaying the risk of diabetes and cardiovascular diseases [4]. Moreover, many studies have shown that changing diet or pharmaceutical intervention can postpone the risk of diabetes in pre-diabetic subjects [5].

Most spices traditionally had medicinal and therapeutic effects on humans but nowadays finding scientific evidence about these effects of spices has become challenging [6]. Several review articles about herbal medicine including spices have been written. Furthermore, spice consumption has been studied for its antioxidant and anti-inflammatory benefits [6, 7]. One of these spices that have many diverse antioxidant agents is cardamom. Cardamom belongs to the ginger family (Zingiberaceae) and its scientific name is *Elettaria cardamomum* [8]. The results of various studies have shown that cardamom flavonoids, which are mainly terpenoids, are responsible for the high antioxidant and medicinal benefits of the spice [6, 9]. They also point out to the fact that flavonoids function in different mechanisms [10].

Limited studies have examined the effects of cardamom on human subjects. One such study showed that cardamom supplementation favourably changed the atherogenic lipid profile—such as LDL, TG, total cholesterol—increased plasma fibrinolytic activity, and improved serum total antioxidant status [11]. Other clinical trial showed that cardamom supplementation significantly reduced blood pressure and improved serum total antioxidant status in subjects with hypertension [12]. However, only a few studies have been carried out on the benefits of cardamom consumption by humans, and the effect of this spice has not been studied in subjects with pre-diabetes. Thus, this study has been designed to determine the effect of cardamom supplementation on serum lipids, glycemic indices, and blood pressure in overweight and obese pre-diabetic women.

## Methods

### Study design and subjects

A randomized double-blind placebo controlled clinical trial was conducted on 80 overweight and obese pre-diabetic women from February to April 2014. The effects of cardamom supplementation on the anthropometric measures of the subjects have been reported previously [13].

The subjects attended two health centres at Karaj city for diabetes screening. Since those who attended these centres were mostly female, we restricted our study to pre-diabetic women. The subjects were made to sign an informed consent form before they enrolled into the study, which was registered on the Iranian Registry of Clinical Trials website (http://www.irct.ir/, IRCT2014060817254N2).

The sample size of at least 40 in each group by α = 0.05 and the power of 80% was determined according to TG [11]. At least one of these inclusion criteria was a pre-requisite: FBS: 100–125 mg/dl; HbA1C: 5.7–6.4%; 2-h blood glucose: 140–199 mg/dl; age: 30–70 years; diagnosis duration of pre-diabetes at least 2 weeks and at most 2 months; BMI: 25–39.9 kg/m^2^; having at least one of the following criteria: 300 > TG > 150 mg/dl, TC > 200 mg/dl, 160 > LDL-C > 100 mg/dl, HDL-C < 50 mg/dl; and willingness to participate in the study. Exclusion criteria were the following: BMI < 25 or ≥40 kg/m^2^; following a specific diet for the previous 3 months; being a professional athlete; having allergy to cardamom; pregnancy and lactation; nutritional supplement and multi vitamin-mineral consumption at least two times a week in the last month; medical history of gastrointestinal ulcers, kidney or gall stones; medical history of coronary heart diseases, cancer, and multiple sclerosis; consumption of blood lipid, glucose, and pressure lowering drugs; hormonal, thyroid, nervous and heart diseases medications; and having blood pressure > 130/80 mmHg.

It is noteworthy that all pre-diabetic patients usually attend some educational classes for their blood glucose control and nutritional education.

### Randomization and intervention

Stratified randomization based on age and BMI and permuted blocks of random numbers were used for random allocation. Stratified randomization was based on the following assortments: age ≤ 40 years and 41–70 years; BMI: 25–29.9 kg/m^2^ and 30–39.9 kg/m^2^. Randomization was performed by an assistant and the intervention allocation was blinded for the investigator and participants. The participants were randomly assigned into two groups receiving whole green cardamom powder or placebo supplements for 2 months. Cardamom and placebo capsules were prepared by the Traditional Medicine and Materia Medica Research Centre (TMRC)**,** Shahid Beheshti University of Medical Sciences, Tehran, Iran. Each capsule contained 1 g of whole green cardamom or rusk powder. Placebo capsules were quite similar to cardamom capsules by the means of shape, size, and colour. All placebo capsules were placed near the cardamom capsules so that they take on the smell of cardamom. Although both cardamom and placebo capsules appeared identical, it is possible that some of the participants could discern a difference between the two.

The subjects were asked to take three capsules daily with meals. In order to increase the compliance of subjects, weekly phone calls were made to them. Further, the women were asked to bring the bottles of supplement back on their last visit, and compliance was evaluated by counting the remaining capsules of the bottles. Subjects with less than 90% of supplement intake were excluded from the study.

**Fig. 1:**
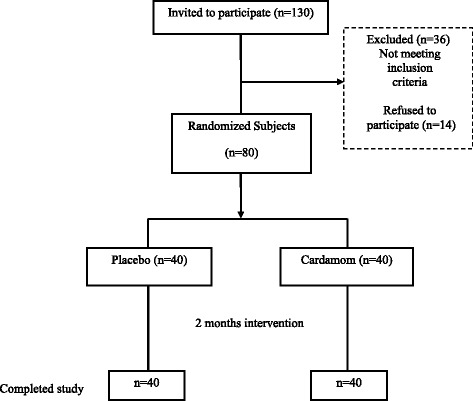
Selection of subjects for participation in the study

The 3 g dose used in the study was chosen based on the ethno medicinal recommendation and other studies [11, 12]. Moreover, 3 g of green cardamom would be a large but not unreasonable amount to consume as a part of the diet. The participants were requested to follow their usual diet and physical activity throughout the intervention. The timing and subjects selection are shown in Fig. 1.

### Dietary analysis

Dietary intake was estimated using a 24-h food recall at the beginning and at the end of the study. USDA food composition table and Nutritionist software version 4 (First Data Bank, San Bruno, Ca, USA) were employed to estimate the daily dietary intake of nutrients.

### Anthropometry and physical activity assessments

Anthropometric measurements including weight, height, and Waist Circumference (WC) were performed by standard methods. BMI was calculated by dividing weight by the square of height.

A shorter form of the International Physical Activity Questionnaire (IPAQ) was used to assess the physical activity of participants during the previous 7 days [14]. The questionnaire included seven items evaluating vigorous and moderate intensity of activities and walking for at least 10 min/day during the previous week. Based on the physical activity questionnaire, low physical activity means having less than 600 MET-minutes/week; moderate physical activity means having less than 600–3000 MET-minutes/week; and intense physical activity means having more than 3000 MET-minutes/week scores.

### Blood pressure and laboratory measurements

Systolic and diastolic blood pressure was measured using a mercury sphygmomanometer (Microlife, Switzerland) before and after the intervention in fasting state. After 12 h of fasting, 10 ml blood sample was taken from patient and FBS, TG, TC, LDL, HDL, and serum insulin were measured before and after the study.

The homeostasis model assessment-estimated insulin resistance **(**HOMA**-**IR**)** index was calculated according to the following formula: [insulin (μU/ml)* Fasting glucose (mmol/L)] /22.5 [15]. On the other hand, the quantitative insulin-sensitivity check index (QUICKI) was calculated using the following formula: [1/ (log glucose_0_ + log insulin_0_)] [16].

### Statistical analysis

The normality of the distribution of data was first checked by the one-sample Kolmogorov-Smirnov test, and the differences between qualitative variables were assessed using the Chi Squared test. While Paired t-test and Wilcoxon were used to compare pre-and post-intervention variables within the groups considering the normality of data. Further Student’s-t and Mann-Whitney tests were carried out to compare groups when they were normally or abnormal distributed, respectively. Analysis of covariance (ANCOVA) was used to identify any differences between the two groups after adjustment for the baseline measurements and covariates. All the analyses were performed using SPSS version 16 (SPSS Inc., Chicago, IL, USA). Differences with a *P* < 0.05 were considered to be statistically significant.

## Results

All participants completed the study, and in terms of compliance all participants consumed 90–100% of the prescribed supplements.

Participants were assessed for any side effects of supplements intake. The reported side effects in the cardamom group included diarrhoea (*n* = 2) in the first few days of study, mild inflammation of skin (*n* = 1) and glossitis (*n* = 1) in the first week of study, and reducing of burning foot (*n* = 10) during the study. In addition, one subject in the placebo group reported constipation in the first few days of study.

### General characteristics, dietary intakes, and anthropometric measurements

There were no significant differences between general characteristics and pre-diabetes diagnosis duration in both groups at baseline. The physical activity levels of the two groups were also similar before and after the intervention (Table 1).

**Table 1:** Characteristics of green cardamom and placebo groups

	Cardamom group (*n* = 40)		Placebo group (*n* = 40)		*p*-value
	Mean or N	SD or %	Mean or N	SD or %	
Age (years)	48.3	10.4	47.5	10.3	0.7^a^
pre-diabetes diagnosis duration (weeks)	5.3	2.04	5.8	1.8	0.2^a^
^a^Physical Activity level					
Before					
low	32	80	30	75	0.5^b^
medium	8	20	10	25	
total	40	100	40	100	
After					
low	32	80	29	72.5	0.4^b^
moderate	8	20	11	27.5	
total	40	100	40	100	

^a^Low physical activity: score less than 600 MET-minutes/week and medium physical activity: score 600–3000 MET-minutes/week

^a^Independent sample t-test

^b^Chi square test

After intervention, the percentage of energy provided by protein (*p* = 0.03) and the Saturated Fatty Acid (SFA) (*p* = 0.004) intake significantly increased, and poly- (*p* = 0.003) and mono- (*p* = 0.002) unsaturated fatty acids intake and percentage of energy provided by fat (*p* = 0.01) decreased in the cardamom group. In the placebo group, the percentage of energy provided by carbohydrate (*p* < 0.001) and protein (*p* = 0.01) significantly increased, and that of energy provided by fat (*p* < 0.001) decreased after intervention (Table 2).

**Table 2:** Comparison of dietary intake before and after the intervention between the two groups

		Before		After		*p*-value ^a^	*p*-value ^b^
		Mean	SD	Mean	SD		
Energy (Kcal)	Cardamom	2107.5	317.04	2153.4	198.9	0.1	0.9
	Placebo	2157.7	242.2	2179.3	215.1	0.5	
	*p*-value^c^	0.4		0.5			
Carbohydrate (%)	Cardamom	49.8	7.06	51.6	5.5	0.09	0.6
	Placebo	48.1	6.6	51.3	4.8	<0.001	
	*p*-value^c^	0.2		0.7			
Carbohydrate (g)	Cardamom	262.1	52.5	277.9	39.9	0.01	0.6
	Placebo	258.8	41.1	279.5	38.1	0.003	
	*p*-value^c^	0.7		0.8			
Protein (%)	Cardamom	10.2	1.7	11.08	1.9	0.03	0.2
	Placebo	9.7	1.6	10.8	2.09	0.01	
	*p*-value^c^	0.2		0.6			
Protein (g)	Cardamom	53.7	11.9	59.8	12.9	0.005	0.9
	Placebo	52.1	8.7	59.06	12.6	0.006	
	*p*-value^c^	0.4		0.7			
Fat (%)	Cardamom	36.1	7.5	33.1	6.1	0.01	0.9
	Placebo	38.2	7.05	33.9	5.4	<0.001	
	*p*-value^c^	0.1		0.5			
Fat (g)	Cardamom	84.8	21.9	79.2	16.05	0.09	0.9
	Placebo	92.1	21.5	82.3	15.8	<0.001	
	*p*-value^c^	0.4		0.3			
Cholesterol (mg)	Cardamom	108.1	94.02	100.8	75.03	0.1	0.1
	Placebo	85.2	62.1	87.9	51.9	0.2	
	*p*-value^d^	0.2		0.6			
Dietary fiber (g)	Cardamom	15.3	9.9	16.2	8.5	0.2	0.07
	Placebo	13.04	6.9	13.07	5.5	0.9	
	*p*-value^c^	0.2		0.053			
Saturated fatty acid (g)	Cardamom	18.6	5.9	21.7	4.3	0.004	0.004
	Placebo	18.3	5.04	19.1	3.7	0.3	
	*p*-value^c^	0.8		0.005			
Mono unsaturated fatty acids (g)	Cardamom	36.4	10.6	32.8	9.5	0.002	0.9
	Placebo	40.7	10.3	34.5	8.08	0.058	
	*p*-value^c^	0.07		0. 4			
Poly unsaturated fatty acids (g)	Cardamom	29.7	10.5	24.6	7.4	0.003	0.06
	Placebo	33.04	10.2	28.6	7.9	0.4	
	*p*-value^c^	0.1		0.02			

^a^Paired t-test

^b^Analysis of covariance, adjusted for baseline values

^c^Independent sample t-test

^d^Mann-Whitney test

The comparison between the two groups showed that after intervention, SFA (*p* = 0.005) intake was significantly higher and Poly Unsaturated Fatty Acid (PUFA) (*p* = 0.02) intake was lower in the cardamom group. However, after adjusting for baseline values, only SFA difference remained significant (*p* = 0.004) (Table 2).

The comparison between the two groups showed that after adjusting for baseline values, cardamom supplementation significantly decreased waist circumference values (*p* = 0.03). There were no significant differences between intervention and control groups in mean weight and BMI (Table 3).

**Table 3:** Comparison of anthropometric indices before and after the intervention between the two groups

		Before		After		*p*-value ^a^	*p*-value ^b^
		Mean	SD	Mean	SD		
Weight (kg)	Cardamom	76.06	11.6	75.6	11.7	0.01	0.06
	Placebo	73.7	10.7	73.7	10.8	0.7	
	p-value^c^	0.3		0.4			
Body mass index (kg/m2)	Cardamom	29.7	4.04	29.5	4.01	0.01	0.06
	Placebo	29.3	3.1	29.04	3.1	0.8	
	*p*-value^c^	0.6		0.8			
Waist circumference (cm)	Cardamom	100.7	8.1	100.2	8.1	0.06	0.03
	Placebo	100.2	7.1	100.4	7.1	0. 2	
	*p*-value^c^	0.7		0.8			

^a^Paired t-test

^b^Analysis of covariance, adjusted for baseline values

^c^Independent samples t-test

^d^Mann-Whitney test

### Effect of cardamom supplementation on blood pressure and blood biomarkers

After intervention, mean TC (*p* = 0.02) and LDL-C (*p* = 0.01) significantly decreased and insulin sensitivity (*p* = 0.03) increased in the cardamom group. In addition, in the cardamom group, mean HDL-C levels were maintained while in the control group, there was a significant decrease after the study (*p* = 0.02) (Table 4).

**Table 4:** Comparison of glycemic indices, serum lipids and blood pressure before and after the intervention between the two groups

		Before		After		*p*-value ^a^	*p*-value ^b^
		Mean	SD	Mean	SD		
Fasting blood glucose (mg/dl)	Cardamom	110.1	9.6	108.1	12.8	0.2	0.9
	Placebo	102.5	8.8	102	9.2	0.6	
	*p*-value^d^	<0.001		0.01			
Insulin (μIU/ml)	Cardamom	18	8.07	17.02	8.3	0.3	0.6
	Placebo	15.6	7.7	15.3	12.3	0.056^c^	
	*p*-value^e^	0.1		0.1			
Insulin sensitivity (QUICKI)	Cardamom	0.30	0.01	0.31	0.02	0.03	0.5
	Placebo	0.31	0.01	0.32	0.02	0.1	
	*p*-value^d^	0.02		0.055			
Insulin resistance (HOMA-IR)	Cardamom	89.3	44.9	81.6	40.7	0.2	0.5
	Placebo	72.3	39.3	71.04	67.7	0.06^c^	
	*p*-value^e^	0.02		0.04			
Triglyceride (mg/dl)	Cardamom	167.9	69.9	150.9	49.1	0.056	0.4
	Placebo	159.1	75	151.8	65.1	0.4	
	*p*-value^d^	0.5		0.9			
Total cholesterol (mg/dl)	Cardamom	192.6	33.5	183.7	37.9	0.02	0.1
	Placebo	194.4	37.7	193.6	33.7	0.8	
	*p*-value^d^	0.8		0.2			
LDL-C (mg/dl)	Cardamom	118.1	25.8	110.5	29.6	0.01	0.1
	Placebo	117.9	22.9	117.1	23.6	0.8	
	*p*-value^d^	0.9		0.2			
HDL-C (mg/dl)	Cardamom	44.1	8.9	42.7	9.4	0.1	0.9
	Placebo	45.3	11.2	43.2	9.8	0.02	
	*p*-value^d^	0.6		0.8			
LDL-C / HDL-C	Cardamom	2.7	0.7	2.6	0.8	0.4	0.2
	Placebo	2.7	0.7	2.8	0.7	0.4	
	*p*-value^d^	0.9		0.5			
Systolic blood pressure (mmHg)	Cardamom	115.5	12.9	115.7	13.08	0. 7	0.8
	Placebo	120.7	13.7	120.5	13.1	0.6	
	*p*-value^d^	0.08		0. 1			
Diastolic blood pressure (mmHg)	Cardamom	77.5	5.8	77.1	5.1	0.4^c^	0.059
	Placebo	78.1	6.1	78.7	5.8	0.1	
	*p*-value^e^	0.6		0. 2			

^a^Paired t-test

^b^Analysis of covariance, adjusted for SFA intake changes and baseline values

^c^Wilcoxon

^d^Independent samples t-test

^e^Mann-Whitney test

At the beginning of study, mean QUICKI in the placebo group was more than the intervention group (*p* = 0.02). In the intervention group mean FBS and HOMA-IR were significantly higher than the placebo group before and after the study (*p* < 0.05) (Table 4).

After adjusting for SFA intake changes and baseline values, we observed no significant decrease in systolic and diastolic blood pressure, glycemic indices, and serum lipids values in the cardamom group compare to placebo group (Table 4).

## Discussion

This study is one of the few randomized clinical trials evaluating the effects of green cardamom supplementation on serum lipids, glycemic indices, and blood pressure in overweight and obese pre-diabetic women. Our results showed that compared to placebo, an eight-week cardamom supplementation does not affect blood pressure and blood biomarkers. However, TC and LDL-C significantly decreased and insulin sensitivity increased in the cardamom group. Furthermore, in the cardamom group, mean HDL-C levels were maintained while in the control group, there was a significant decrease after the study, suggestive of a protective effect of cardamom on HDL-C level. It might be possible that cardamom proves beneficial for subjects with more blood parameters disturbances. Moreover, different results may be obtained by a higher dose of supplement and a longer duration of intervention.

Although comparison between the two groups has not shown statistically significant differences, there was a greater reduction of some blood parameters in the intervention group compared to the placebo group. The reduction was seen in TG (−10 vs. -4.6%), TC (−4.6 vs. -0.4%), and LDL-C (−6.4 vs. -0.7%), which could reflect the effects of cardamom intake. These results were observed despite the significant increase of SFA and decrease of PUFA intake in the cardamom group. In addition, compared to the beginning of the study, mean serum HDL-C decreased in both groups. However, this reduction was statistically significant only in the placebo group, which could have been prevented by cardamom intake in the intervention group. However, HDL-C reduction in the control group might be the result of the decrease of total fat intake during the study.

We have previously reported that compared to the placebo group, at the end of the study, WC in the intervention group decreased significantly [13]. The effects of cardamom on blood lipids might also be related to its effect on WC.

So far, there have been only two clinical trials that studied the effects of cardamom supplementation in humans. Verma’s study reported that consumption of 3 g cardamom powder for 12 weeks by people with hypertension (stage 1) significantly decreased systolic and diastolic blood pressure [12]. But in our study, even after 2 months of 3 g cardamom powder supplementation, systolic and diastolic blood pressure did not show any significant improvement. The discrepancy between our data and that shown in the mentioned study could be attributed to the shorter intervention duration of our study. In addition, the subjects in Verma’s study were hypertensive patients and it did not have any control group, which might be the reason for showing more benefits of cardamom. Further, Verma’s study did not show any changes in the blood lipids level in the intervention group [12]. Another study by Verma, which was conducted on 30 male patients with ischemic heart disease, showed that consuming 3 g of large cardamom (Amomum subulatum Roxb.) powder for 12 weeks significantly reduced atherogenic blood lipids level, including LDL-C, TG, and TC in the intervention group. Higher content of 1,8-cineole has been accounted for the significant hypolipidemic activity of large cardamom [11].

Pre-diabetic subjects usually have high BMI and WC. In addition, their blood glucose, insulin, and inflammatory proteins are high. Inflammatory proteins might have a role in insulin resistance [17], which is one of the causes of type 2 diabetes, endothelial dysfunction, high blood pressure, and abnormal lipid profile that can eventually lead to heart diseases. Spices can be used as a supportive therapy in the prevention and control of insulin resistance [18]. In fact, some spices have stimulating effect on insulin secretion, which can effectively improve the performance of insulin on lipid metabolism [6]. Furthermore, a large number of spices and herbs are traditionally used to control hyper glycemia. The results of some studies have shown the effects of cinnamon in the control of blood glucose. For example, Jarvil-Taylor et al. showed that cinnamon consumption can activate glucose uptake, glycogen synthesis, and glycogen synthase enzyme in adipocytes [19]. On the other hand, Khan et al. showed that consumption of cinnamon reduced FBS and improved blood lipids in patients with dyslipidemia [20].

The positive properties of cardamom are mainly due to its volatile oil, which has terpene, esters, flavonoids, and other compounds. The major compounds of the oil are 1, 8 cineole (36.3%) and α-terpinyl acetate (31.3%) [21]. 1, 8 -cineole, a monoterpenic oxide, has vascular relaxant, anti-inflammatory, and antioxidant properties [22–24]. In addition, studies show that the health effects of spices are related to their flavonoids component, so similar effects of different spices on blood pressure, glucose indices, and lipid profile can largely be explained by their flavonoids content as well.

It has also been shown that by preventing pancreatic lipase activity, some flavonoids can reduce the absorption of fats [25, 26]. They can directly affect the active site of enzyme or by increasing the size of fat micelles (triglycerides), indirectly reducing access of enzyme to substrate [26, 27]. The result of some studies on patients with metabolic syndrome has shown foods with high flavoniods content reduce serum TG, TC, and LDL-C and increase HDL-C. Moreover, flavoniods have an effect on transcription factors such as proteins, Sterol Regulatory Element-Binding Protein (SREBP-1), and SREBP-2 sterol regulatory element-binding, which increase cholesterol and TG synthesis [28].

The role of flavonoids in the prevention of insulin resistance has also been researched. One of the main factors, which results in insulin resistance in adipose tissue is the increase of fat storage in adipocytes, which leads to inflammation. Flavonoids, by reducing fat storage, might improve insulin function in the body [28].

Our study has some limitations, including a small sample size and a short intervention period. Moreover, male subjects could not be included in the study. Therefore, the findings of this study might not be applicable to all pre-diabetic subjects. Despite these limitations, this study is among the few ones that determine the effect of green cardamom supplementation on the blood pressure and blood biomarkers in overweight and obese pre-diabetic women. Moreover, we used whole cardamom, which, compared to spice extract, might be considered more in line with applied nutritional therapy.

## Conclusion

The present study shows that although green cardamom supplementation improves some blood parameters in pre-diabetic subjects, its effects are not different from placebo. However, cardamom may have a protective effect on HDL-C level. Further research is needed to clarify the effects of green cardamom in pre-diabetic subjects. Study at the cellular level might precisely identify the mode of action of this popular spice.

## Abbreviations

BMI: Body mass index
FBS: Fasting blood sugar
HDL: High density lipoprotein
IPAQ: International physical activity questionnaire
LDL: Low density lipoprotein
MUFA: Mono unsaturated fatty acid
PUFA: Poly unsaturated fatty acid
SFA: Saturated fatty acid
SREBP: Sterol regulatory element-binding protein
TC: Total cholesterol
TG: Triglyceride
WC: Waist circumference
